# Development of an In Vitro Model for the Multi-Parametric Quantification of the Cellular Interactions between Candida Yeasts and Phagocytes

**DOI:** 10.1371/journal.pone.0032621

**Published:** 2012-03-30

**Authors:** Karine Dementhon, Sofiane El-Kirat-Chatel, Thierry Noël

**Affiliations:** 1 Univ. Bordeaux, Microbiologie Fondamentale et Pathogénicité, UMR 5234, Bordeaux, France; 2 CNRS, Microbiologie Fondamentale et Pathogénicité, UMR 5234, Bordeaux, France; University of Minnesota, United States of America

## Abstract

We developed a new *in vitro* model for a multi-parameter characterization of the time course interaction of *Candida* fungal cells with J774 murine macrophages and human neutrophils, based on the use of combined microscopy, fluorometry, flow cytometry and viability assays. Using fluorochromes specific to phagocytes and yeasts, we could accurately quantify various parameters simultaneously in a single infection experiment: at the individual cell level, we measured the association of phagocytes to fungal cells and phagocyte survival, and monitored in parallel the overall phagocytosis process by measuring the part of ingested fungal cells among the total fungal biomass that changed over time. *Candida albicans*, *C. glabrata,* and *C. lusitaniae* were used as a proof of concept: they exhibited species-specific differences in their association rate with phagocytes. The fungal biomass uptaken by the phagocytes differed significantly according to the *Candida* species. The measure of the survival of fungal and immune cells during the interaction showed that *C. albicans* was the more aggressive yeast *in vitro*, destroying the vast majority of the phagocytes within five hours. All three species of *Candida* were able to survive and to escape macrophage phagocytosis either by the intraphagocytic yeast-to-hyphae transition (*C. albicans*) and the fungal cell multiplication until phagocytes burst (*C. glabrata, C. lusitaniae*), or by the avoidance of phagocytosis (*C. lusitaniae*). We demonstrated that our model was sensitive enough to quantify small variations of the parameters of the interaction. The method has been conceived to be amenable to the high-throughput screening of mutants in order to unravel the molecular mechanisms involved in the interaction between yeasts and host phagocytes.

## Introduction

Deep and invasive fungal infections caused by *Candida* species are increasing among immunocompromised individuals. *Candida albicans* is the species most often involved, but emerging non-*albicans* species, such as *C. glabrata*, *C. tropicalis*, *C. parapsilosis*, *C. krusei, C. guilliermondii* and *C. lusitaniae* are becoming increasingly frequent and are responsible for nearly half of candidiasis cases [1]. When infecting the host, the fungal cells are confronted with innate immune cells, essentially macrophages and neutrophils. Given the evidence that phagocytosis of fungal cells is the first step in the control of infection, developing a cellular model allowing an accurate analysis of the overall interaction involving different *Candida* species and phagocytes, appears to be of great interest in that way it constitutes an alternative method to *in vivo* experiments to evaluate virulence of *Candida* strains.

The main objective of this work was to develop a simple and reproducible method for the simultaneous monitoring of the kinetics for phagocyte association to yeasts, phagocyte survival at the individual cell level, and for fungal cells uptake by phagocytes over a 24-hour infection. The second objective was to make the method sensitive enough to detect small variations during the yeast-phagocyte interaction, and amenable to high-throughput screening of banks of mutants. An accurate evaluation of phagocytosis requires 1) to analyze the phagocyte association to yeast cells and the phagocyte survival simultaneously 2) to distinguish between yeasts that had been internalized by phagocytes from those unphagocytosed, and to measure the uptake of fungal cells by phagocytes while taking into account extracellular yeast multiplication during the infection process. A critical step is to label yeasts during the infection process. Most of the studies already reported used FITC (Fluorescein isothiocyanate, a dye that covalently binds amino acids of proteins [2]) to separately label yeasts before infection. However, the use of FITC made the phagocytosis analysis limited to early time points [3], because yeast replication led to fluorescence decrease, or restricted to fixed cells stained with antibodies coupled with FITC for longer incubation time points. Instead, we chose CalcoFluor White (CFW) which specifically labels fungal cell walls [4]: when added to the culture medium, it allowed the continuous labeling of yeasts, even those that are newly generated by replication along the 24-hour infection. Phagocytes were double-stained with calcein, a marker of active metabolism and membrane integrity, and anti-CD16 antibodies which stained the membrane. To determine the rate of yeasts internalized in phagocytes, we exploited the ability of the trypan blue, incapable of penetrating into viable phagocytes [5], to quench the fluorescence of the extra-phagocyte CFW-labeled yeasts, in order to detect solely the CFW fluorescence of the internalized yeasts [6].

Microscopy is commonly used to count the number of phagocytes associated to yeasts as well as the number of yeasts internalized within phagocytes, or cell survival using vital dye exclusion [7]–[8]. However, microscopy may be too cumbersome for analyzing a large number of strains. Several studies described the use of flow cytometry or fluorometry to measure the association of the phagocytes to the yeasts, and the uptake of yeasts by phagocytes [9], [10], [11], [12], [13], [14], [15]. Therefore, we chose to use flow cytometry and modified a previously published assay [6] for the kinetics studies of the proportion of phagocytes associated to yeasts (either simply attached to the membrane or internalized) and phagocyte survival when infected by yeasts (compared to uninfected phagocytes).

In this work, we describe an improved *in vitro* model of phagocyte infection with yeast, that allows an accurate quantification of both cell types (phagocytes and fungal cells) interaction and outcome during a single infection experiment up to 24 hours, and suitable for high-throughput screening of small phenotypic differences. As a proof of principle, we compared to which extent three species of *Candida*, exhibiting different morphological types, could differ in their interaction with murine macrophages and human neutrophils. We chose *Candida albicans*, which can develop under the form of yeast, pseudo-hyphae and true hyphae, *Candida lusitaniae*, found as yeast and pseudo-hyphae forms, and *Candida glabrata* only existing as a unicellular yeast form. We demonstrated that our model was sensitive enough to point out differences of interaction between those three *Candida* species and two types of phagocytic cells, and practical to analyze a very large number of each cell type over a 24-hour interaction.

## Results

### Fluorometry and Flow Cytometry Allow an Accurate Quantification of the *in Vitro Candida* - Macrophage Interaction

The challenge in developing an *in vitro* cellular model to perform kinetics studies relied on the fact that the two interacting populations changed over time: the fungal cells grew and divided, leading to an overall increase in the fungal biomass, and the infected phagocytes eventually died, imposing the need to specifically mark each population to monitor their outcome during the interaction. Accordingly, the fungal cells were continuously labeled with Calcofluor White (CFW) which binds chitin and glucans in the cell wall of both live and dead yeast [16]. Because of the morphological changes that could occur for the different yeast species during the infection process, variations in the CFW fluorescence were interpreted as variations in the fungal biomass rather than variations in the number of cells. The murine macrophages of the J774 cell line were double stained with calcein, a marker of active metabolism and membrane integrity, and anti-CD16 antibodies which stained the membrane; the phagocytes that were fluorescent for both markers were considered to be alive. We checked that CFW, the only stain present during the infection process, did not alter the growth or viability of yeasts and phagocytes at the concentrations used (Figures S1 and S2). Furthermore, using the microscope and trypan blue as a vital dye, we checked that CFW was neutral for macrophages viability, and that alive macrophages, intensely fluorescent for calcein, did not accumulate trypan blue (data not shown).

**Figure 1:**
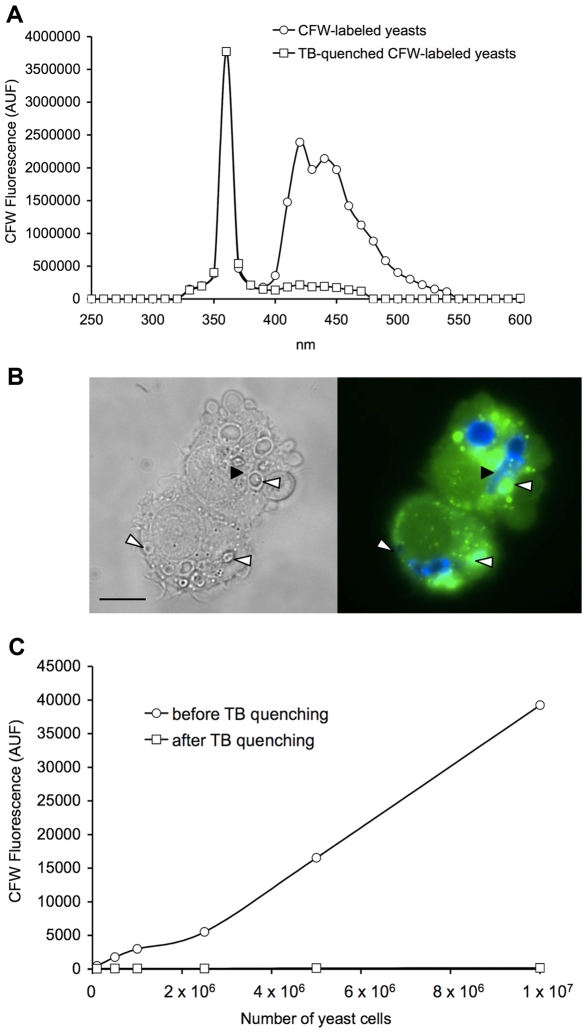
Trypan blue efficiently quenched CFW-labeled yeast fluorescence. (A) Emission spectrum of CFW-labeled yeasts before and after trypan blue (TB) quenching. 1×10^7^ *C. lusitaniae* cells were labeled with 5 µg/ml CFW, and 250 µg/ml trypan blue was used for quenching. Excitation wavelength was set up at 360 nm and emission wavelength was set up to cover the entire emission spectra of CFW. (B) Macrophages after 4 hours of infection with *C. lusitaniae*. Yeast cells were stained with CFW (blue), macrophages were stained with calcein (green). Left panel: phase contrast, right panel: fluorescence. The fungal cells ingested inside the macrophages were protected from trypan blue quenching and were fluorescent, whereas membrane-bound yeasts exposed to trypan blue quenching were not (some examples are shown with white arrowheads). A pseudohyphae of *C. lusitaniae* can be seen within the macrophage (black arrowhead). The bar represents 20 μm. (C) Fluorometric detection of CFW-labeled *C. lusitaniae* cells before and after trypan blue quenching. Trypan blue used at 250 μg/ml quenched the CFW fluorescence to background level for up to 1×10^7^ *C. lusitaniae* cells.

Further controls were performed to determine and validate the experimental conditions that were used to analyze the interaction between the two stained cell types by fluorometry. First, we verified that calcein fluorescence was proportional to the number of macrophages (Figure S3). Moreover, in order to quantify the multiplication of the fungal biomass, total fluorescence of CFW-labeled yeasts was measured in PBS. We established that CFW fluorescence was linear with the OD_600_ up to 24 hours for the three *Candida* species when inocula up to 1×10^6^ CFU per 200 µl in the microplate well were used to infect macrophages. Thus, CFW fluorescence intensity is proportional to the fungal biomass (Figure S4). Accordingly, all infection experiments were performed using yeast inocula of 6×10^4^ CFU or 1×10^5^ CFU depending on the MOI (Multiplicity Of Infection). The trypan blue quenching assay, based on the extinction of the fluorescence of extracellular yeasts (*i.e*, free yeasts and yeasts adhering to phagocytes but not internalized), was used to determine the percentage of the total fungal biomass that was phagocytosed by macrophages. First, we checked that trypan blue efficiently quenched the fluorescence of free CFW-labeled yeasts (Figure 1A): *C. lusitaniae* cells were labeled with 5 µg/ml CFW. CFW emits a fluorescence around 430–460 nm when bound to the yeast cell wall and gives negligible background fluorescence when unbound. When 250 µg/ml trypan blue was added to the CFW-labeled yeasts, the CFW fluorescence was quenched to the baseline observed for CFW alone. Using the microscope, we confirmed the quenching of the membrane-bound CFW-labeled yeasts (Figure 1B). Then, we determined the concentrations of trypan blue that were necessary to quench the CFW fluorescence emitted by up to 1×10^7^ yeast cells of the three species used in this study : 250 µg/ml of trypan blue was sufficient to quench the CFW fluorescence of both *C. lusitaniae* and *C. glabrata*, whereas 1 mg/ml was needed for *C. albicans* (*e.g.* Figure 1C for *C. lusitaniae*).

**Figure 2:**
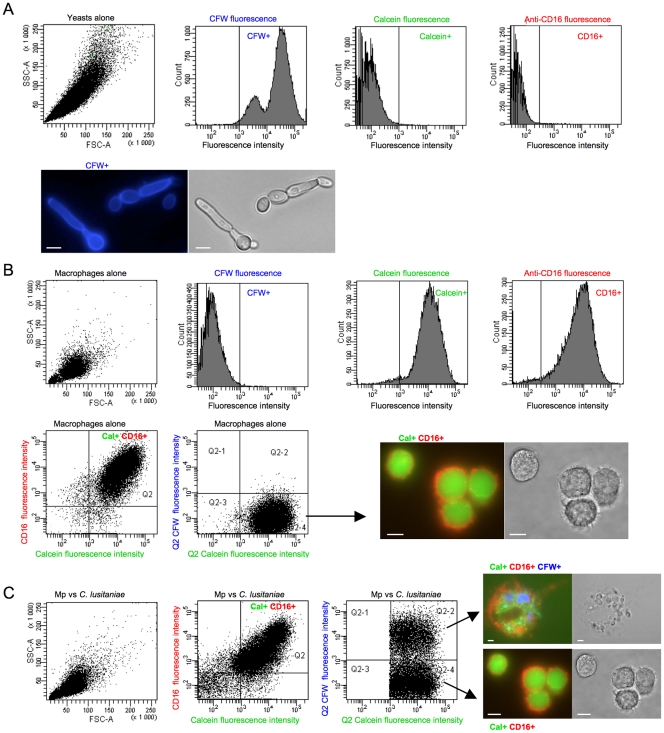
Flow cytometry analysis of the macrophages interacting with yeasts. *C. lusitaniae* cells and macrophages were stained as described in the Materials and Methods. Histograms plot the cell size (SSC) against cellular complexity (FSC) or the number of cells against the fluorescence intensity. The vertical lines define the baseline above which the fluorescence is positive, and was gated using each labeled population alone, and unstained cells as negative controls. Thus, the CFW-labeled yeast cells alone only showed a positive signal for CFW fluorescence (A), the calcein and anti-CD16 double-stained macrophages alone only showed a positive signal for calcein and anti-CD16 fluorescences (B). The anti-CD16 fluorescence was plotted against the calcein fluorescence to gate the population Q2, corresponding to alive double-stained calcein+anti-CD16+macrophages. When the calcein and anti-CD16 double-stained macrophages were infected with CFW-labeled yeasts (C), the analysis of the population Q2 for CFW fluorescence distribution after 5 hours of incubation showed two distinct cells subsets: Q2-2 corresponding to phagocytosing macrophages, and Q2-4 corresponding to non-phagocytosing macrophages. The scale bars on the microscopy panels represent 5 µm (A and upper panel in C) or 30 µm (B and lower panel in C).

We used flow cytometry in order to accurately quantify the association of macrophages with the fungal cells (in other words the percentage of phagocytosing macrophages) and assess the viability of the phagocytes. The CFW-labeled yeasts and the anti-CD16 and calcein double-stained macrophages were observed as distinct populations on the flow cytometer (Figure 2A and 2B). When infected with yeasts (Figure 2C), two distinct subsets of macrophages could be distinguished among the alive macrophages (quadrant Q2): the macrophages associated with yeasts (membrane-bound and/or ingested), *i.e* phagocytosing macrophages, positive for CFW fluorescence (quadrant Q2-2), and non-phagocytosing macrophages, negative for CFW fluorescence (quadrant Q2-4). The different populations were sorted at T 5h and observed with the microscope to verify that the cells subset that was positive for calcein, anti-CD16 and CFW fluorescence truly corresponded to the macrophages that had taken up the yeast cells (Figure 2C). The percentage of macrophage viability was calculated as the number of macrophages positive for both fluorescence (calcein and anti-CD16) in an infection assay *versus* the uninfected macrophages control. The term “aggressiveness” was used to qualify the effect of the yeast cells on macrophage viability.

### Macrophages are Less Efficient to Control *Candida Albicans* than *C. Lusitaniae* or *C. Glabrata* Infection in Both the Stationary and Exponential Phase at a MOI of 1M:1Y

**Figure 3:**
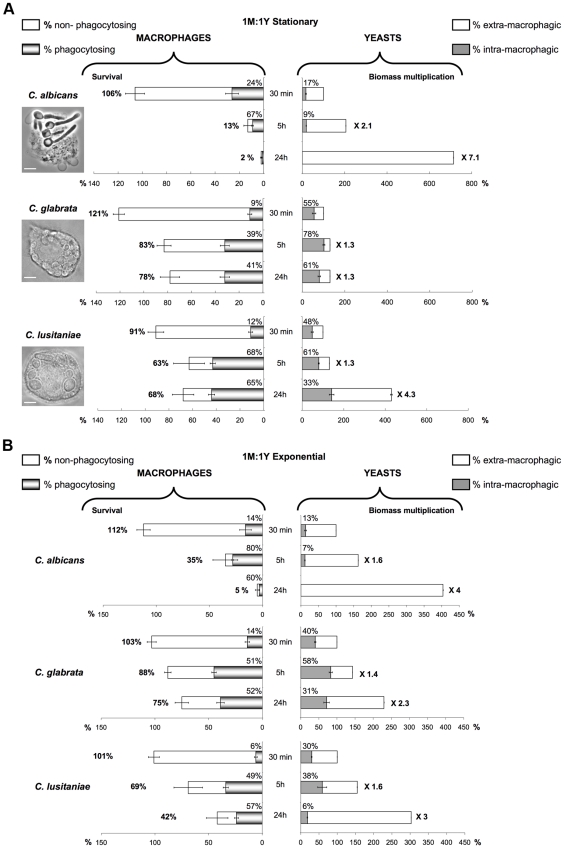
Analysis of the interactions involving the J774 macrophages and living yeast cells in stationary and exponential phases over 24-hour time course experiments. The left part of the diagram shows the flow cytometry analysis of the J774 macrophages: J774 survival percentage is indicated on the left side of the horizontal bar. The white area of the bar indicates the percentage of non-phagocytosing macrophages, the shaded tones area indicates the percentage of phagocytosing macrophages. The right part shows the fluorometry analysis of the fungal population: the increase in the biomass compared to T 30 min is given as a multiplication factor on the right side of the horizontal bar. The white area of the bar represents the percentage of extra-macrophagic yeasts, the grey area shows the percentage of fungal biomass internalized within the J774 macrophages (*i.e* the uptake of fungal biomass). Interactions of J774 macrophages with stationary phase (A) or exponential phase (B) living yeasts at 1M:1Y MOI. Note that macrophage killing and the part of phagocytosing macrophages were higher with *C. albicans* than with *C. glabrata* and *C. lusitaniae* (T 5 h and T 24 h), and that *C. albicans* uptake was lower compared to the two other species. Each condition was performed in quintuplet (flow cytometry experiments) or in triplicate (fluorescence quenching experiments) per experiment. Each bar is the average of three independent experiments ± standard error. See also Figure S5 which details how this diagram was built. The scale bars on the light microscopy panels represent 10 µm for the upper and middle panels and 5 µm for the lower panel.

It is known that the yeast cell wall composition and transcript profile vary with the physiological state of development [17], notably when considering cells grown in the stationary *vs.* exponential phase. The J774 macrophages were infected with *C. albicans*, *C. lusitaniae* and *C. glabrata* blastospores from either the exponential or stationary phase at a MOI of 1M:1Y and the phenotypes of the interactions were compared over time. Results are represented in Figure 3 (see also Figure S5), showing in parallel the behavior of the macrophages (percentage of viable macrophages and percentage of phagocytosing macrophages), and the behavior of the yeast cells (increase of the total fungal biomass and percentage of engulfed fungal biomass) during the infection process. After 30 min of incubation, nearly 100% of the macrophages infected with the different yeast species were still alive. Occasionally, we observed a slight increase of the number of active macrophages when infected macrophages were compared to uninfected controls, probably due to an increased metabolism in response to the presence of yeasts. Overall, few macrophages were engaged in phagocytosis of yeast cells as early as 30 min (6 to 24% according to the yeast species).

*C. albicans* appeared to be the more aggressive species in terms of macrophage killing (Figure 3A and 3B, left bars): only 13% of the macrophages survived after five hours of infection with stationary yeast cells; interestingly, macrophages survival was higher at T 5 h with exponential yeast cells. However, nearly all macrophages were completely destroyed after 24 h of incubation. The process of killing was dependent upon the formation of hyphae by *C. albicans* that pierced the majority of the macrophages (Figure S6, Movie S1). On the other hand, *C. albicans* was less internalized by the macrophages than the two other species (Figure 3A and 3B, right bars): only 7 to 17% of the fungal biomass was engulfed according to the duration of incubation. No fungal cells were detected in viable macrophages after the fluorescence quenching experiment, indicating that the entire *C. albicans* population was unengulfed at T 24 h**.** Thus, the majority of the cells multiplied outside macrophages, as verified with the microscope (Figure S6), leading to an important fungal biomass development (x 7 or x 4 at T 24 h).

Survival of macrophages was much higher when infected with *C. glabrata* (nearly 80% survival at T 5 h and T 24 h), with up to 40 to 50% macrophages involved in phagocytosis (Figure 3A and 3B, left bars). There was no difference in macrophage killing between stationary and exponential yeasts. The proportion of macrophages engaged in phagocytosis with *C. glabrata*, as well as the proportion of viable macrophages, were similar at both T 5 h and T 24 h, indicating that *C. glabrata* did not kill the macrophages and that the proportion of macrophages engaged in phagocytosis reached a plateau. The rate of yeasts engulfed was greater for *C. glabrata* than for *C. albicans* (Figure 3A and B, right bars). The proportion of the *C. glabrata* yeasts whose fluorescence was unquenched (78%, Figure 3A, T 5 h, right bar) suggested that the vast majority of the cells were engulfed by intact macrophages**.** Using a microscope, we confirmed that all of the *C. glabrata* cells derived from stationary phase were cleared out from the medium by the J774 macrophages at T 5 h (Figure S6). The rate measured with fluorometry did not reach 100% probably because some killed macrophages allowed a portion of the engulfed fungal cells to be quenched. *Candida glabrata* yeast cells remained intra-macrophagic at 24 hours post-infection as indicated by the observation with the microscope and by the fact that the total CFW fluorescence did not increase between T 5 h and T 24 h. The CFW can not enter viable macrophages [18], accordingly, it was not possible to determine if the fungal cells multiplied inside the J774 macrophages.

The multiplication of the *C. glabrata* fungal biomass was restricted (x 1.3) when stationary yeast cells were used, and was slightly higher when exponential yeast cells were used (up to x 2.3 at T 24 h). We could verify with the microscope that this increase in fungal biomass was derived from the extra-macrophagic multiplication of unengulfed yeasts, whereas the stationary yeast cells were kept fully ingested by the phagocytes (Figure S6). Furthermore, when using *C. glabrata* yeasts taken from exponential rather than stationary phase, 10% more macrophages were recruited for phagocytosing 20% less fungal cells (see the data obtained for T 5 h for example). Altogether, these results suggested that the *C. glabrata* yeasts derived from exponential phase were less efficiently recognized by the phagocytes.

Globally, *C. lusitaniae* behaved similarly to *C. glabrata* with however some interesting differences of interaction with macrophages. Survival of macrophages infected with *C. lusitaniae* was generally 10% lower than with *C. glabrata,* except at T 24 h when using exponential phase yeast cells, which resulted in a marked decrease of macrophage survival down to 42% (Figure 3B, left bars). Phagocytosis of *C. lusitaniae* also required a higher number of macrophages when compared to *C. glabrata*: 65% of the live macrophages were engaged in phagocytosis of *C. lusitaniae* stationary yeast cells, and 50–55% for exponential yeast cells. The proportion of engulfed yeast cells was generally lower with *C. lusitaniae*, and the proportion of free extra-macrophagic yeasts was higher than with *C. glabrata*. We verified with the microscope that *C. lusitaniae* was never cleared off the medium by the macrophages (Figure S6). Yeasts divided actively outside leading to an increase of the fungal biomass at T 24 h (x 4.3 with stationary phase cells, and x 3 with exponential phase cells, of which 94% were extra-macrophagic). These results indicated that the *C. lusitaniae* cells were less efficiently internalized by the J774 macrophages than the *C. glabrata* cells.

By comparing the data obtained at T 5 h and T 24 h with stationary yeast cells of *C. lusitaniae*, it is interesting to note that the increase of intra-cellular fluorescence (Figure 3A, right bars) was not correlated with an increase of the number of phagocytosing macrophages which remains constant (left bars); this suggested that the macrophages that had already ingested fungal cells were more susceptible to engulf other yeast cells than naïve macrophages. The analysis of the data obtained in the same conditions with *C. lusitaniae* exponential yeast cells (Figure 3B) led to a completely different conclusion: the 30% decrease in macrophage survival from T 5 h to T 24 h can be set in parallel with a considerable decrease of the engulfed fungal biomass (from 38% to 6%, meanwhile the biomass was only multiplied by x 2), suggesting that part of the internalized *C. lusitaniae* had escaped macrophages.

By assaying the glucose available in the culture medium during the infection process (Method S1 and Table S1), we verified that macrophage cell death observed during infection was neither due to any nutritional starvation nor to a release of a toxic compound in the supernatant by one of the cell types. These results indicated that J774 macrophage mortality was directly related to the phagocytosis of the *Candida* cells. A relationship could be drawn between the glucose consumption and the outcome of the interaction at T 5 h: when yeast cells were in the majority engulfed, the glucose consumption was similar to that of the macrophages alone, whereas when the yeast cells were extra-macrophagic, the consumption of glucose resembled that of the yeasts alone. When infected with UV-killed yeasts, macrophages used 2.5 times more glucose than uninfected macrophages. This reflects the metabolic cost of phagocytosis of dead fungal cells.

On the other hand, we investigated the capacity of the macrophages to kill the different species of *Candida*. The survival of the yeast cells was determined following 5 and 24 hours of incubation with phagocytic cells (Method S2 and Figure S7). 100% of the *C. albicans* cells survived, while 20% and 40% of the *C. glabrata* and *C. lusitaniae* cells were killed within 24 hours of interaction with the macrophages, respectively.

In conclusion, the J774 macrophages were less efficient in phagocytosing *C. albicans* compared to *C. glabrata* and *C. lusitaniae*. Our results also suggested that the exponential phases of *C. lusitaniae* and *C. glabrata* escaped more easily from macrophage phagocytosis than the stationary phase cells. Even if the growth-dependent physiological state of the *Candida* cells influenced the interaction with the macrophages at some levels (macrophage survival or the efficiency of the fungal biomass uptake), the global profile of the interaction within a *Candida* species was similar for the exponential and stationary phases. Thus, for technical convenience, we chose to infect the phagocytes with stationary cultures in the subsequent experiments. Our choice to work with cells in the stationary phase was reinforced by a study that showed that the transcriptional profile of yeast cells *in vivo* during an infection episode was related to the stationary phase in the laboratory [19].

### Interaction of Inactivated Yeast Cells with Macrophages

**Figure 4:**
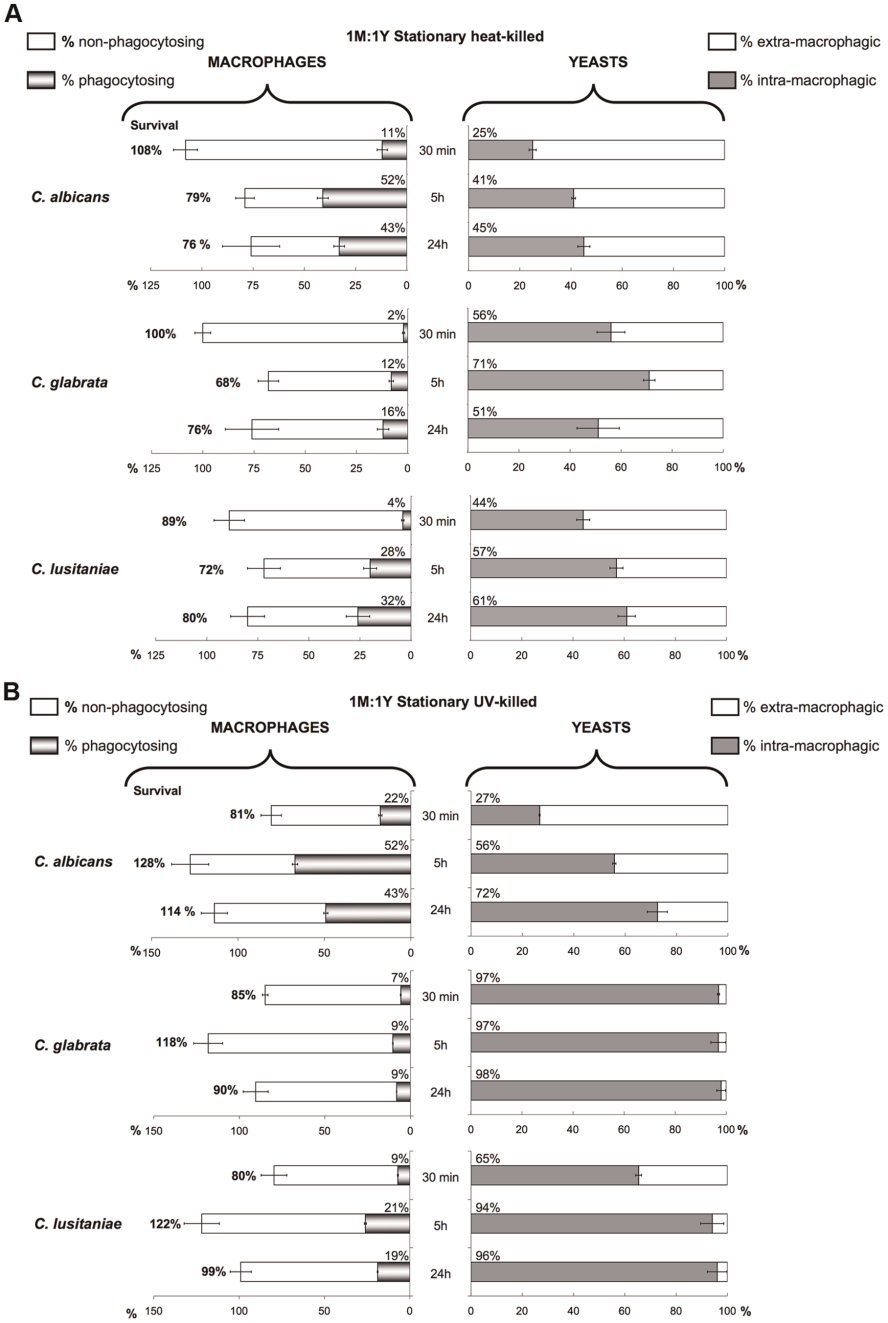
Effect of heat-killing and UV-killing treatments of the fungal cells on the interactions with the J774 macrophages. Analysis of the interactions involving J774 macrophages and stationary-phase heat-killed (A) or UV-killed (B) yeasts over 24-hour time course experiments. Note that the yeasts uptakes were higher for UV-killed cells. For the legend description, please refer to Figure 3.

We first inactivated yeasts with heat treatment. When we used heat-killed (HK) yeasts, we observed about 20% of macrophages cell death at T 24 h when compared to uninfected macrophages controls, independently of the species used (Figure 4A). The J774 macrophages were the most efficient at taking up *C. glabrata* dead cells (16% of phagocytosing macrophages internalized 51% of the fungal biomass at T 24 h) and the least efficient at taking up *C. albicans* dead cells (43% of phagocytosing macrophages internalized 45% of the fungal biomass). The comparison of live and inactivated cells in terms of the efficiency of phagocytosis by the macrophages is only relevant at T 30 min, before yeasts multiplication takes place. The amounts of macrophages phagocytosing HK yeasts were smaller than with the live cells, independently of the species used. On the other hand, the fungal biomass internalized was similar for HK yeasts and live yeasts (20 to 55% depending on the species). The decrease in the amount of macrophages engaged in phagocytosis could be attributed to the loss of the fungal metabolic activity and/or to the cell wall alterations resulting from heat treatment.

To determine if the manner of killing yeasts had an effect on the interaction with the J774 macrophages, we also infected phagocytes with UV-killed yeasts (Figure 4B). UV-killed cells lose their ability to replicate but retain an intact cell wall [20]. The amounts of macrophages engaged in phagocytosis with UV-killed yeasts were higher than when heat-killed yeasts were used, and similar to the live cells, suggesting a predominant role of the cell wall in the early recognition process. Furthermore, the amount of internalized fungal biomass was significantly increased for UV-killed cells compared to live cells (between 30% and nearly 100% depending on the species were engulfed at T 30 min). One may suppose that live cells produce a signal molecule (which would be inactivated by UV treatment) that interferes with the recognition by the macrophages. Moreover, the inactivation of the yeast cells confirmed some inter-species differences already noticed with live cells in terms of macrophage recognition: the J774 macrophages were the most efficient at taking up *C. glabrata* dead cells and the least efficient at taking up *C. albicans* dead cells.

Live *C. glabrata* and *C. lusitaniae* cells triggered a slightly increased macrophage mortality compared to when inactivated (compare Figure 3A and Figure 4A and B). These data suggest a little contribution of fungal active mechanisms for both *C. glabrata* and *C. lusitaniae* in the macrophage killing. In contrast, live *C. albicans* cells triggered a much higher macrophage mortality (approximately 85% of macrophage killing after 5 hours and nearly 100% after 24 hours) than when inactivated (20% maximum). These data suggest that the macrophage cell death induced by *C. albicans* involves fungal active mechanisms.

### Using *C. Lusitaniae* and *C. Glabrata* Cells in Excess Increased the Macrophage Cell Death and Escape from Phagocytosis

**Figure 5:**
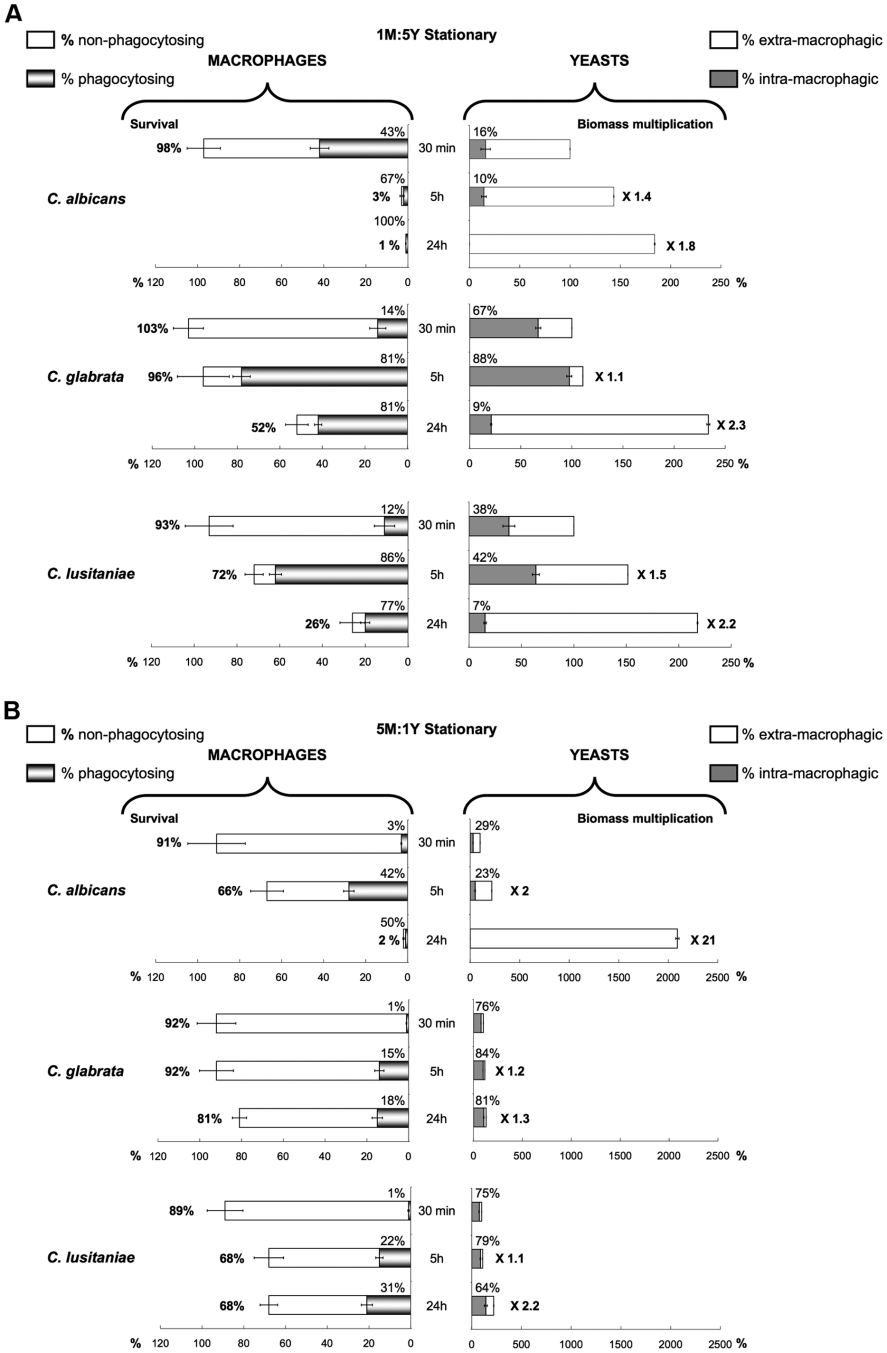
Effect of the MOI on the interactions between J774 macrophages and yeasts over 24-hour time course experiments. (A) Interactions of J774 macrophages and stationary-phase living yeasts at 1M:5Y MOI. Note that more macrophages died with *C. glabrata* and *C. lusitaniae* than at 1M:1Y MOI at T 24 h. Again the highest part of phagocytosing macrophages and macrophage cell death rates were obtained with *C. albicans*. Note the higher uptake of *C. glabrata* cells, and the escape from macrophages of the three *Candida* species at T 24 h. (B) Interactions of J774 macrophages and stationary-phase living yeasts at 5M:1Y MOI. Note that the macrophage killing with *C. albicans* was delayed compared to 1M:1Y MOI. Note the equally high uptake of *C. glabrata* and *C. lusitaniae* and the lower uptake of *C. albicans* biomass, that was the only one species that finally escaped the macrophages at that 5M:1Y MOI. For the legend description, please refer to Figure 3.

**Figure 6:**
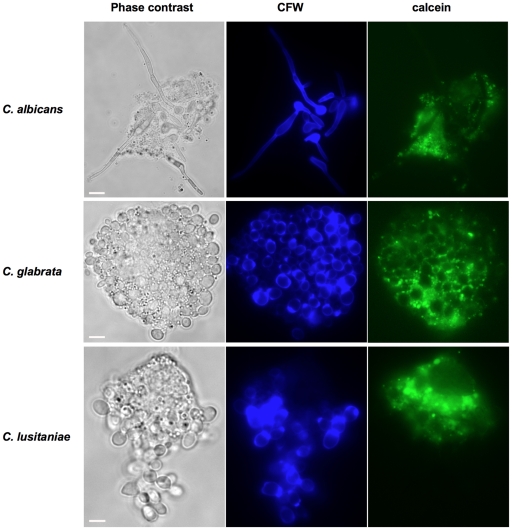
*C. albicans*, *C. glabrata* and *C. lusitaniae* escaping from macrophages. Macrophages were observed with the microscope after 5 hours of infection with *C. albicans* and 24 hours of infection with *C. glabrata* and *C. lusitaniae.* Yeast cells were stained with CFW (middle panel), macrophages were stained with calcein (right panel). See also video-microscopy of the interaction between J774 macrophages and *C. albicans* at a MOI of 1M:1Y (Movie S1), *C. glabrata* at a MOI of 1M:5Y (Movie S2) and *C. lusitaniae* at a MOI of 1M:2Y (Movie S3) over a 24-hour incubation period. The scale bar represents 5 µm.

Challenging the macrophages with an excess of yeasts at a MOI of 1M:5Y affected macrophage survival. As expected, *C. albicans* was more aggressive than the other two *Candida* species at T 5 h. Interestingly, the macrophages infected with an excess of *C. glabrata* or *C. lusitaniae* showed an increased mortality compared to a MOI of 1M:1Y at T 24 h (48% *vs.* 22% in *C. glabrata* infections and 74% *vs.* 32% in *C. lusitaniae* infections; Figure 3 and Figure 5A). Despite the fact that the macrophages were equally engaged in phagocytosis with *C. glabrata* and *C. lusitaniae* (approximately 80% of live macrophages for both species at T 5 h and T 24 h), the proportion of internalized biomass differed significantly (88% of *C. glabrata* were taken up at T 5 h *vs.* 42% for *C. lusitaniae* (Figure 5A), again indicating that the macrophages were less efficient at engulfing *C. lusitaniae* compared to *C. glabrata.* At T 24 h, only 9% of the *C. glabrata* biomass and 7% of the *C. lusitaniae* biomass were within the macrophages. Together with the increase in macrophage killing, this observation suggested that both *C. glabrata* and *C. lusitaniae* escaped the macrophages after 24 hours of infection at a MOI of 1M:5Y, causing phagocyte death, and multiplied outside the macrophages. We confirmed by observation under the microscope and video-microscopy that the three *Candida* species were capable of escaping from macrophage phagocytosis (Figure 6 and Movies S1 - S3). At the beginning of the infection, *C. albicans* yeasts were phagocytosed by macrophages. After 30 minutes, yeasts started to form hyphae even inside macrophages. Hyphae disrupted the phagocyte membrane after 2–3 hours and allowed yeast to escape phagolysis (Movie S1). We observed that *C. glabrata* cells were massively phagocytosed by the J774 macrophages, but survived and multiplied inside, leading to phagocyte burst (Movie S2). Interestingly, we observed that *C. lusitaniae* cells were phagocytosed at the beginning of the infection, but after 2 hours, started to form chains of cells that were no longer recognized by phagocytes: macrophages got in contact with yeast chains but rarely engulfed them, which allowed yeasts to multiply extracellularly (Movie S3).

### When Macrophages are used in Excess, the Killing of Macrophages by *C. Albicans* is Delayed and *C. Glabrata* and *C. Lusitaniae* are More Efficiently Taken Up

When used in excess at a MOI of 5M:1Y, the macrophages were able to better survive *C. albicans* infection at T 5 h than at a MOI of 1M:1Y (66% *vs.* 13% survival; Figure 3 and Figure 5B), but after 24 hours, 98% of the phagocytes were killed and the entire fungal biomass was extra-macrophagic. Compared to a MOI of 1M:1Y, when the phagocytes were used in excess, smaller percentages of macrophages were engaged in phagocytosis with the yeast cells and higher proportions of fungal biomass were ingested, independently of the *Candida* species used. Only 1% of the live macrophages took up approximately 80% of the *C. glabrata* and *C. lusitaniae* biomass at T 30 min (Figure 5B). *Candida glabrata* remained fully contained until T 24 h, whereas *C. lusitaniae* multiplied outside of the macrophages, as observed with the microscope. These results showed that when macrophages are used in excess with respect to yeasts, they more efficiently resist *C. albicans* induced-cell death during the early stages of interaction and, as expected, engulf more fungal biomass, irrespective of the *Candida* species used.

### *C. Albicans* Triggers a Higher Neutrophil Cell Death than *C. Glabrata* and *C. Lusitaniae*

We analyzed the interaction of the three *Candida* species with human neutrophils at different MOIs, using the same procedure as described above for the macrophages, except that quenching experiments could not be realized for a technical reason: the neutrophils were loosely adherent to plastic, compared with the macrophages, and were lost during the washes that were required for the trypan blue quenching. We did not try further to artificially make the neutrophils adhere to the bottom of the well using gelatin or collagen in order to avoid the interference of an external molecule with the *Candida-*phagocyte interaction. Furthermore, the determination of the percentage of internalized yeast cells is a less discriminatory parameter with neutrophils because a non-negligible portion of the neutrophils are able to capture and destroy pathogens extracellularly owing to the formation of NETs (Neutrophils Extracellular Traps) [21].

**Figure 7:**
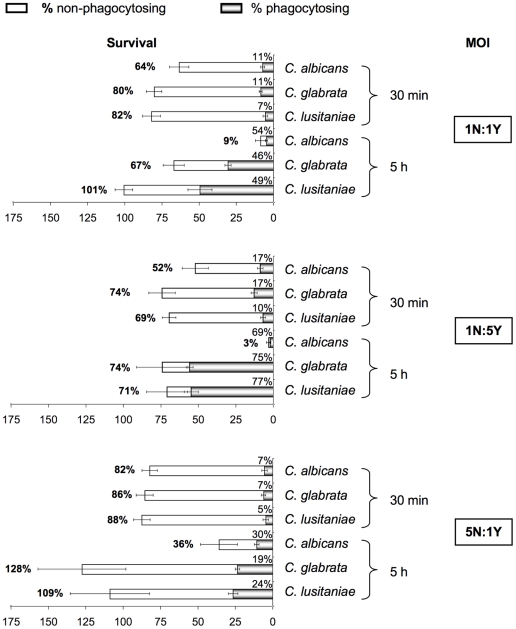
Analysis of human neutrophil interaction with live yeast cells in stationary phase at different MOIs over 5-hour time course experiments. The diagram shows the flow cytometer analysis of the neutrophils. The horizontal bar represents the neutrophil survival, indicated as a percentage on the left side of the bar. The white part of the bar represents the percentage of non-phagocytosing neutrophils, the shaded tones part represents the percentage of phagocytosing neutrophils. Note that more neutrophils died when infected with *C. albicans* than with *C. glabrata* and *C. lusitaniae*. Each condition was performed in quintuplet per experiment. Each bar is the average of three independent experiments ± standard error.

We first measured the survival of the neutrophils and their association with the live stationary phase fungal cells by flow cytometry over a 5-hour infection period with a MOI of 1N:1Y (Figure 7). After 30 minutes, 20% of the neutrophils died in the presence of *C. lusitaniae* and *C. glabrata*, whereas 40% died in the presence of *C. albicans*. A longer infection time did not significantly change the killing of neutrophils when infected by *C. lusitaniae* and *C. glabrata*, but dramatically increased the killing by *C. albicans* (more than 90% of the neutrophils were killed at T 5 h). The association between the neutrophils and the three *Candida* species was similar during the infection and reached 50% at T 5 h.

Increasing the number of yeasts over that of the neutrophils (MOI 1N:5Y) resulted in an approximate 10% increase in neutrophil cell death for each *Candida* species in the early stages of the infection (Figure 7), as approximately 30% the of neutrophils were killed by *C. glabrata* and *C. lusitaniae*, whereas 50% were killed by *C. albicans.* Nearly all of the neutrophils were killed by *C. albicans* within a 5-hour period. Furthermore, as expected, the neutrophils were more associated with the fungal cells at a MOI of 1N:5Y compared to a MOI of 1N:1Y during the infection. In the experiments conducted at a MOI of 5N:1Y, it is noteworthy that the neutrophils survived infection by *C. albicans* as well as the other species at T 30 min, and 36% were still alive at T 5 h. Also, as expected, the neutrophils were less associated with the yeast cells than they were at MOIs of 1N:1Y and 1N:5Y. In conclusion, while the association of the neutrophils with the three *Candida* species was similar and varied proportionally with the MOIs, neutrophil cell death was both similar and moderate in the presence of *C. glabrata* and *C. lusitaniae. Candida albicans* induced a higher neutrophil cell death, which varied proportionally with the MOIs.

## Discussion

This study aimed to develop an improved *in vitro* model to finely quantify the cellular interactions between *Candida* yeasts and phagocytes. The method we developed is the first one that allows a semi-automated multi-parameter analysis for the simultaneous monitoring of two interacting populations in a host-pathogen context. The method was validated with three different *Candida* species and two types of phagocytes, the J774 murine macrophage cell line and human neutrophils.

Yeasts were labeled with CFW, a fluorochrome that binds cell wall of live and dead fungal cells. CFW shows a relative insensitivity of its fluorescence to pH, which is an advantage in a phagocytic assay as the phagolysosomal acidification can modify the fluorescence of an internalized yeast [22]. The CFW was added in the culture medium at the beginning of the infection process to allow the continuous labeling of newly replicated yeasts. An increase in CFW fluorescence over time was interpreted as an increase in the fungal biomass, independently of the morphology and size of the cells. As the CFW can not enter viable macrophages, the multiplication of yeasts within macrophages was not assessed, unless yeasts were released in the medium. Phagocytes were specifically labeled with calcein-AM and anti-CD16-APC antibodies. Calcein-AM is a fluorogenic esterase substrate, used as a viability probe that reflects both enzymatic activity, which is required to convert calcein-AM to a fluorescent product, and cellular membrane integrity, which is required for intracellular retention of the fluorescence. Calcein-AM was chosen because cell viability studies showed it was quite well retained by living cells and released during cytolysis, with low pH sensitivity [23]. Anti-CD16-APC antibodies specifically labeled the membrane of phagocytes.

To avoid the counting with the microscope, we chose flow cytometry to analyse the population of phagocytes. This method allows a rapid analysis of a large number of cells, and a simultaneous measure of two parameters at the individual cell level: the percentage of phagocytes engaged in phagocytosis of yeasts (either bound on the phagocyte membrane or internalized), and the survival of that same population of infected phagocytes, compared to their survival in absence of yeasts. To differentiate the CFW-labeled yeasts that were free or simply bound to the macrophage membrane from those ingested, we used the trypan blue to quench the CFW fluorescence of extra-macrophagic yeasts, leaving the internalized yeasts fluorescent.

From a strict analytical point of view, our model overcomes a bias in the interpretation of the data frequently generated when the percentage of phagocytosing macrophages and the percentage of internalized fungal biomass are analyzed separately. For example, our data showed that the percentage of phagocytosing macrophages was lower for *C. glabrata*, which could be interpreted as a lower recognition efficiency, but the percentage of internalized fungal biomass was actually higher for *C. glabrata* than for the other *Candida* species. Also, a lower percentage of internalized fungal cells does not necessarily reflect a lower internalized fungal biomass, as extra-macrophagic fungal cells may divide over time, and increase total fungal biomass. Thus, our model takes into account not only the percentage of phagocytosing macrophages, along with the macrophage survival, but also the percentage of total fungal biomass internalized.

When we compared the three *Candida* species, we observed differences in the proportion of macrophages engaged in phagocytosis, in the survival rate of macrophages and in the efficiency of the macrophages to internalize the fungal biomass. We established that macrophages were more efficient to phagocyte *C. glabrata*, as indicated by the lowest number of macrophages engaged in phagocytosis, the lowest macrophage cell death, and the highest proportion of the fungal biomass internalized. *C. albicans* was the more aggressive species as it triggered the highest number of macrophages engaged in phagocytosis, the highest macrophage cell death and the lowest proportion of internalized fungal biomass. We found that *C. lusitaniae* was more efficiently taken up than *C. albicans*, and in smaller amounts than *C. glabrata*. The differences in the aggressiveness of the *Candida* species toward the phagocytes highlighted in our work correlate with already published studies, especially those showing that *C. glabrata* was considerably less virulent than *C. albicans* using a mouse model [24], and *in vitro* competition experiments showing that J774 macrophages display strong preferences for phagocytosis of *C. glabrata* compared to *C. albicans* [25] .

The differences in the interaction of the three *Candida* species with the macrophages may be in part attributed to a different cell wall composition, whose role in the recognition by the phagocytes has been widely described [8], [15], [26], [27], [28], [29], [30]. It has been reported that the cell wall of *C. glabrata* harbors 50% more mannose and three times less chitin than *C. albicans* [31]. As the mannans are exposed on the external face of the cell wall, they are likely to be more easily recognized by the receptors (Mannose Receptor, TLR4, Dectin-2, Galectine-3) of the phagocytes, whose activation initiates phagocytosis [29]. Furthermore, the cell wall composition can vary depending on the developmental state of the fungal cells [17]. *Candida albicans* shows a differential expression of cell wall proteins in yeast and hyphae [32], [33], [34], including particular glycosyltransferases that modify the glycan components, and therefore the PAMPs (Pathogen-Associated Molecular Patterns) exposed on the cell surface. Also, hyphae of *C. albicans* do not expose beta-glucans like yeast cells do in bud scars, and thus do not activate the Dectin-1 receptor, which is involved in phagocytosis and immune response processes [35]. Beta-glucans of *C. lusitaniae* may be similarly masked in the small chains of cells, thus explaining why under that morphology, it is less recognized by the macrophages.

Our experiments showed that the growth phase of the fungal cells is important for the host-pathogen interaction. When yeast cells were taken from the exponential phase, less *C. lusitaniae* and *C. glabrata* biomass was engulfed by the macrophages and they escaped more easily from macrophage than when taken from the stationary phase. We speculated that some cell wall components may be differentially exposed such as beta-1,2 mannosides, recognized by galectin 3 of macrophages, which are in higher amount in the cell wall of yeast from the stationary phase than from the exponential phase [36].

The experiments conducted with inactivated yeast cells allowed us to investigate the interaction independently from the morphological changes and from the metabolic activity of the yeast cells. Heat treatment suppresses metabolic activity but artificially increases β-glucan exposure, altering the outer layer of mannans, whereas UV treatment was shown to maintain an intact cell wall [20]. We found that macrophages were more efficient to internalize UV-killed than heat-killed cells, emphasizing a predominant role of the cell wall (and likely of the mannans) in the recognition by the macrophages. Interestingly, UV-killed yeasts were more efficiently internalized by the macrophages than live cells, despite a similar amount of macrophages engaged in phagocytosis. We speculate that live fungal cells produce a signal molecule that negatively interferes with macrophage recognition.

Macrophage cell death was slightly higher when macrophages were infected by living *C. glabrata* and *C. lusitaniae* cells than when infected with dead yeasts: thus we speculated that the fungal metabolic activity of these two species did not significantly contribute to the observed macrophage killing. Up to 20% of the macrophages died in the presence of the inactivated yeast cells, irrespective of the species used. This suggests that the phagocytosis of inert yeast cells triggered macrophage killing *via* a macrophage-dependent mechanism. It has already been reported that the uptake of yeast polysaccharides led to a macrophage cell death of 10–20% after 6 or 24 hours of incubation [37]. In contrast, the ability of *C. albicans* to kill macrophages was greatly reduced after the inactivating treatment. Our data confirm already published studies showing that the metabolic activity of *C. albicans*, and the capacity to produce hyphae, largely contributed to macrophage killing.

In our work, the three *Candida* species mostly survived to macrophage phagocytosis, according to three different mechanisms. As previously shown [38]–[39], our data confirmed that *C. albicans* survived to phagolysis and rapidly produced hyphae from within the macrophages and thus escaped, killing the host cell and multiplying outside. In agreement with other work [40], we found that *C. glabrata* mostly survived phagolysis and divided within the macrophages, which eventually burst and released the yeast cells. Beside its ability to form pseudo-hyphae, we found that *C. lusitaniae* quickly formed small chains of cells, less efficiently recognized by the macrophages as confirmed by video-microscopy. Once phagocytosed, a proportion of the *C. lusitaniae* cells were able to survive, multiply within the macrophages and escape. Thus the present data support the hypothesis that there may be a correlation between the morphology of the *Candida* specie and the strategy used to escape from the macrophages.

Some mechanisms important for the survival and escape of yeasts from phagocytes have recently been described. For *C. albicans,* beside the known yeast-to-hyphae transition [41], the contribution of other mechanisms allowing survival and resistance to phagocyte killing was reported: the inhibition of the phagosome maturation [38], the production of CO_2_ from arginine to induce germ tube formation [42], the trehalose biosynthetic pathway [43], [44] and the expression of Hyr1p, a GPI-anchored cell wall protein of the hyphal form [45], and degradation of host-derived reactive oxygen species (ROS) by fungal superoxyde dismutases [46]. In *C. glabrata,* autophagy [47] and glycosylphosphatidylinositol-linked aspartyl proteases [40] were shown to be crucial for survival within macrophages. The strategies employed by *C. lusitaniae* to survive and escape macrophage phagocytosis remain to be investigated at the molecular level.

Neutrophils are thought to be the most efficient phagocytic cells to fight fungal cells [48] Recognition of yeast cells are mediated by specific PRR, in particular TLR 2, TLR 4 and dectin-1, which recognize the glucans and mannans of the fungal cell wall [29]. It was also shown that phagocytosis by human neutrophils can be elicited solely by β-1,6-glucans [49]. They kill pathogens intracellularly in the phagolysosome by a set of enzymes and anti-microbial molecules, and by the production of ROS [50]–[51]. Alternatively, they can kill pathogens extracellularly, through the degranulation and release of antimicrobial molecules, and the release of Neutrophil Extracellular Traps (NETs) from the dying neutrophils [21], [52]. On the other hand, the pathogens can trigger neutrophil cell death, either by inducing NETs release [53], or by inducing the phagocytosis of the complement or IgG opsonized targets through the CR3 receptor, which leads to neutrophil apoptosis (Phagocytosis-Induced Cell Death or PICD) [54]–[55]. In our experiments, the neutrophils did not significantly differ in their association with the three *Candida* species. However, *C. albicans* triggered the highest neutrophil cell death, regardless of the MOI tested, while *C. glabrata* and *C. lusitaniae* were relatively inefficient to counteract an attack by the neutrophils. As the *Candida* cells were non-opsonized in our experiments, it is likely that the neutrophil cell death we observed was not PICD, but rather due to the release of NETs [21], as it could be observed in our experiments with propidium iodure staining (data not shown).

The *in vitro* cellular model we developed in this study was proved to be sensitive enough to detect phenotypic differences not only between different *Candida* species, but also within a same *Candida* species, differences resulting from small variations in MOI, or from the developmental state of the yeast. The data obtained by our team (unpublished data) also showed that different mutants of *C. lusitaniae* had measurable differences in their interaction with macrophages and neutrophils. Finally, we believe that our model is suitable for large scale screening of banks of *C. lusitaniae* mutants and of other yeast species, with the goal to identify new fungal genes important for each step of the interaction with immune host cells: recognition and ingestion of yeasts by the phagocytes, survival of both cell types, and escape of yeasts from phagocytes.

## Materials and Methods

### Strains, Media and Growth Conditions

*Candida albicans* SC5314, *Candida glabrata* ATCC 90030 and *Candida lusitaniae* CBS 6936 (ATCC 38533) were used as wild-type strains in all of the experiments. The *Candida* strains were grown in standard medium YPD (1% yeast extract, 2% bactopeptone, 2% glucose). The murine macrophage cell line J774A.1 (ATCC TIB-67) was cultured in DMEM (Gibco) containing 10% decomplemented fetal bovine serum (FBS, Gibco) and 1 mM sodium pyruvate, or in complete RPMI (cRPMI: RPMI-1640 (Sigma) without phenol red and supplemented with 10% decomplemented FBS, 1 mM sodium pyruvate and 2 g/L sodium bicarbonate) for the infection experiments, at 37°C in 5% CO_2_. For the preparation of the heat-killed yeasts, the cells were incubated for 30 min at 90°C.

### Isolation and Purification of Human Neutrophils

The neutrophils polymorphonuclear (PMN) cells were isolated and purified from fresh human blood (EFS Aquitaine Limousin, Bordeaux) as described [56]. Briefly, dextran allowed the sedimentation of red blood cells. A hypotonic lysis in the presence of ammonium chloride was used to remove any remaining red blood cells. The leucocytes were deposited onto a Percoll (Fluka, SIGMA) density gradient (1.075/1.095) and centrifuged at 3000×g for 20 min at 4°C. The lower ring containing the PMN was collected, the PMNs were washed with PBS and resuspended in 0.5X cRPMI. The resulting population contained at least 95% neutrophils.

### Infection of Phagocytes with Yeasts

J774 macrophages were plated in culture-treated white 96-well plates with clear well bottoms (Greiner Bio-one) in 200 µl of cRPMI and incubated overnight at 37°C in 5% CO_2_ to adhere. Triplicates or quintuplets of the wells were done in each plate and three plates were set up to perform a time course analysis of the infection over 24 hours (at T 30 min, T 5 h and T 24 h). To prevent variation over time by repeatedly sampling the content of a single plate, a separate plate was set up for each time point. We used 2×10^5^ macrophages per well for two multiplicities of infection (MOI 1M:1Y and 1M:5Y) and 3×10^5^ macrophages per well at a MOI of 5M:1Y. The loss of unadhered cells was lower than 2% of the initial population. Yeast cells were collected from overnight culture in YPD supplemented with 5 µg/ml Calcofluor White (CFW, Sigma) and adjusted to the required concentrations in cRPMI plus 5 µg/ml CFW. Because the size or the morphology of the cells in a culture can influence the value in the OD_600_ measurements, we established the correlation between OD_600_ and CFU for each strain of each yeast species. J774 macrophages were infected with 200 µl of CFW-labeled yeasts in 5 µg/ml CFW at the required concentration depending on the MOIs tested. As a control, the uninfected phagocytes were stained with 5 µg/µl CFW in cRPMI. PBS alone was used as a negative fluorescent control. Yeasts in cRPMI without phagocytic cells were included in the plate. We verified that 5 µg/µl CFW did not alter the growth or viability of phagocytes and yeast cells. The neutrophils were purified just before being infected with yeasts, as described for macrophages, except that the interactions were not followed beyond 5 hours since these phagocytes have a short life span (about 8 hours).

### Fluorometry and Quenching Analysis

CFW fluorescence (λ_ex_ 350 nm, λ_em_ 460 nm) was measured using a FluoStar Optima fluorimeter (BMG Labtech). The results were recorded in arbitrary units of fluorescence (AUF). The vital dye trypan blue (Sigma) was used to quench fluorescence in the spectral range of CFW. The fluorescence of yeasts either free in the medium or attached to the macrophage membrane was quenched. As trypan blue cannot enter viable cells, the unquenched fluorescence reflected the yeast cells that were internalized in viable macrophages. At each time point, the plate was centrifuged 5 min 2200×g and the wells were washed with PBS to remove the unbound CFW. To measure the overall process of phagocytosis, the non-ingested yeasts were not removed during the assay. For the quenching experiments, each condition was duplicated in the plate: 200 µl of PBS was added to one well in order to determine the total fluorescence of the CFW-labeled yeasts (intra-macrophagic and extracellular), and 200 µl of trypan blue was added to another well at a final concentration of either 1 mg/ml for *C. albicans* or 250 µg/ml for both *C. lusitaniae* and *C. glabrata* in order to assess the fluorescence of the internalized CFW-labeled yeasts. After washing the wells with PBS, 200 µl of PBS was added and the CFW fluorescence was measured. For each well, the value of the fluorescence after quenching was compared to the total fluorescence in PBS in order to determine the relative amount of yeasts internalized over time. Uninfected macrophages were treated in the same manner as the infected ones to assess the residual fluorescence not attributable to the yeasts before and after trypan blue quenching. Yeasts alone were included in the plate and treated in the same way in order to validate the efficiency of trypan blue quenching. To follow the multiplication of the fungal biomass in the presence of macrophages along the infection, we compared the total CFW fluorescence values at T 5 h and T 24 h to the initial value at T 30 min and determined the biomass multiplication factors.

### Flow Cytometry Analysis

The quantification of the attachment and/or ingestion of the yeasts by the phagocytic cells was done by flow cytometry analysis using a FACSCanto II (Becton Dickinson) equipped for Calcofluor White (λ_ex_ 365 nm, λ_em_ 430 nm,), calcein-AM (λ_ex_ 496 nm, λ_em_ 516 nm) and anti-CD16-APC (λ_ex_ 600 nm, λ_em_ 630 nm) fluorescence measurements. The phagocytic cells were infected by CFW-labeled yeasts as described above. After different periods of incubation, the plate was kept on ice to stop phagocytosis. Supernatants containing free yeasts were eliminated, and after a PBS wash and trypsin treatment, the phagocytic cells were labeled with 0.2 µg/ml anti-mouse CD16-APC (Beckman Coulter) and 0.2 µM calcein-AM (Sigma) and the samples in the 96-well plates were analyzed by flow cytometry. A constant volume (60 µl) of each sample was measured at a high flow rate (2 µl/s). The data were collected using a linear representation for the side scatter (SSC) and forward scatter (FSC) and a logarithmic representation for the fluorescent signals. The data were then analyzed using the FACSDiva software from Becton Dickinson. As negative controls, the yeast cells alone were labeled with anti-CD16-APC and calcein-AM, and the phagocytic cells alone were labeled with CFW and used to determine the background of each fluorescent marker.

### Microscopy

Aliquots of phagocytosing macrophages and neutrophils were deposited onto glass slides and observed with a Zeiss Axioplan microscope. The images were recorded with a Micromax camera (Princeton Instruments). The wells of the plate were observed using a Zeiss Axiovert 200 microscope and the images were recorded with a Axiocam ICm1 camera (Zeiss).

### Video-microscopy

2×10^5^ phagocytes were plated in 9 cm^2^ Petri dishes with glass bottoms (Iwaki). 4×10^5^ yeast cells in 2 ml of cRPMI medium were added to the phagocytes to start the infection. The movies were recorded at the BIC (Bordeaux Imaging Center), using an inverted video-microscope (Leica) equipped with a QuantEM camera. A recording was done every two minutes for 6 to 9 hours at 37°C under 5% CO_2_, at five different positions on the plate. Z steps were carried out (2 µm step on a range of 14 µm) for each recording. The images were analyzed using the Metamorph Offline software (Molecular Devices).

### Statistical analysis

The differences presented in the Results section were all tested for significance using ANOVA and *t* tests. Differences were considered significant when *P* values were < 0.05.

## Supporting Information

Figure S1 **Yeast cells multiplication in cRPMI medium with and without CFW (5** **µg/ml) by OD_600nm_ measurements.** C.a: *C.albicans*, C.g: *C.glabrata*, C.l: *C.lusitaniae.*
(TIF)

Figure S2 **Macrophages viability in cRPMI medium with and without CFW (5** **µg/ml) by calcein fluorescence measurements.** Each bar is the average of two experiments ± standard error.
(TIF)

Figure S3 **Calcein fluorescence varies proportionaly with the number of macrophages.** Each bar is the average of three experiments ± standard error.
(TIF)

Figure S4 **Yeast cells muliplication over time by OD_600nm_ and CFW fluorescence measurements in cRPMI medium with 5** **µg/ml of CFW.** Dashed lines show linear regression lines, and their slopes are indicated.
(TIF)

Figure S5 **Analysis of the interactions involving the J774 macrophages and stationary-phase living yeast cells at 1M:1Y MOI over 24-hour time course experiments.** Figure S5 details how the diagram of Figure 3A was built. Figure S5A shows the flow cytometry analysis of the macrophages and corresponds to the left part of the diagram of Figure 3A. Each bar represents the viability of infected macrophages compared to uninfected macrophages, and the numbers indicate the parts of phagocytosing (shaded tones) or non-phagocytosing (white tones) macrophages. Note that *C. albicans* engaged the higher part of macrophages in phagocytosis (T 30 min) and killed more macrophages (T 5 h and T 24 h) than *C. glabrata* and *C. lusitaniae*. Figure S5B shows the fluorometry analysis of the fungal cells and corresponds to the right part of the diagram of Figure 3A. Each bar represents the percentage of the total fungal biomass internalized in viable macrophages. Note the lower uptake of *C. albicans* cells. Each condition was performed in quintuplet (A) or in triplicate (B). Each bar is the average of three independent experiments ± standard error.
(TIF)

Figure S6 **Representative pictures of the J774 macrophages after 5 hours of infection with the three** ***Candida*** **species in culture flasks at 1M:1Y MOI.** Note that the totality of *C. glabrata* cells were engulfed, whereas *C. albicans* (mostly in filamentous form) and *C. lusitaniae* cells were still observed outside the macrophages. The scale bars represent 30 µm. See also Movie S1 showing the interaction of J774 macrophages with *C. albicans* at a MOI of 1M:1Y over a 5-hour incubation period.
(TIF)

Figure S7 **Macrophage fungicidal activity toward the different** ***Candida*** **species.** To investigate the capacity of the macrophages to kill the different species of *Candida*, the survival of the yeast cells was determined following 5 and 24 hours of incubation with phagocytic cells at a 1M:1Y MOI (Method S2). 100% of the *C. albicans* cells survived, while 20% and 40% of the *C. glabrata* and *C. lusitaniae* cells were killed within 24 hours of interaction with the macrophages, respectively. *C. a: C. albicans, C. g: C. glabrata, C. l: C. lusitaniae.*
(TIF)

Method S1 **Method involved in glucose assay.** See Table S1.
(DOC)

Method S2 **Method documenting the survival of ingested yeasts.** See Figure S7.
(DOC)

Movie S1 **Video-microscopy observation of J774 macrophages infected with** ***C. albicans*** **at 1M:1Y MOI.**
(AVI)

Movie S2 **Video-microscopy observation of J774 macrophages infected with** ***C. glabrata*** **at 1M:5Y MOI.**
(AVI)

Movie S3 **Video-microscopy observation of J774 macrophages infected with** ***C. lusitaniae*** **at 1M:2Y MOI.**
(AVI)

Table S1 **Macrophage cell death is not due to a nutritional depletion of the medium during infection.** The quantity of glucose available was measured in the supernatant of the J774 macrophages that were infected with live or UV-killed yeasts of the three *Candida* species for 5 and 24 hours at a MOI of 1M:1Y (Method S1), and compared to uninfected macrophages, yeasts alone and fresh cRPMI alone containing 2 g/L glucose. The uninfected macrophages depleted approximately 40% of the glucose present in the media after 24 hours. When the macrophages were infected with the fungal cells, nearly 100% of the glucose was depleted after 24 hours, independently of the species used. After 5 hours, a higher glucose depletion was observed when the J774 cells were infected with *C. albicans*. Interestingly, *C. albicans* and *C. glabrata* alone used about 70–75% of the glucose at T 5 h, whereas *C. lusitaniae* used only 40%.
Next, we checked if this glucose depletion could be responsible for macrophage mortality during infection (data not shown). The macrophages were incubated for five hours with the different supernatants collected from infection experiments and filter-sterilized, before assessing their survival by flow cytometry. Despite the total depletion of glucose in the media after 24 hours of infection, none of the supernatants tested triggered macrophage cell death. These results indicated that J774 macrophage mortality was directly related to the phagocytosis of the *Candida* cells.
(DOC)
