# Supplementary figures and images for: Development of an In Vitro Model for the Multi-Parametric Quantification of the Cellular Interactions between Candida Yeasts and Phagocytes
Source: PLoS One. 2012 Mar 30;7(3):e32621. doi: 10.1371/journal.pone.0032621 (PMC3316538; doi:10.1371/journal.pone.0032621)

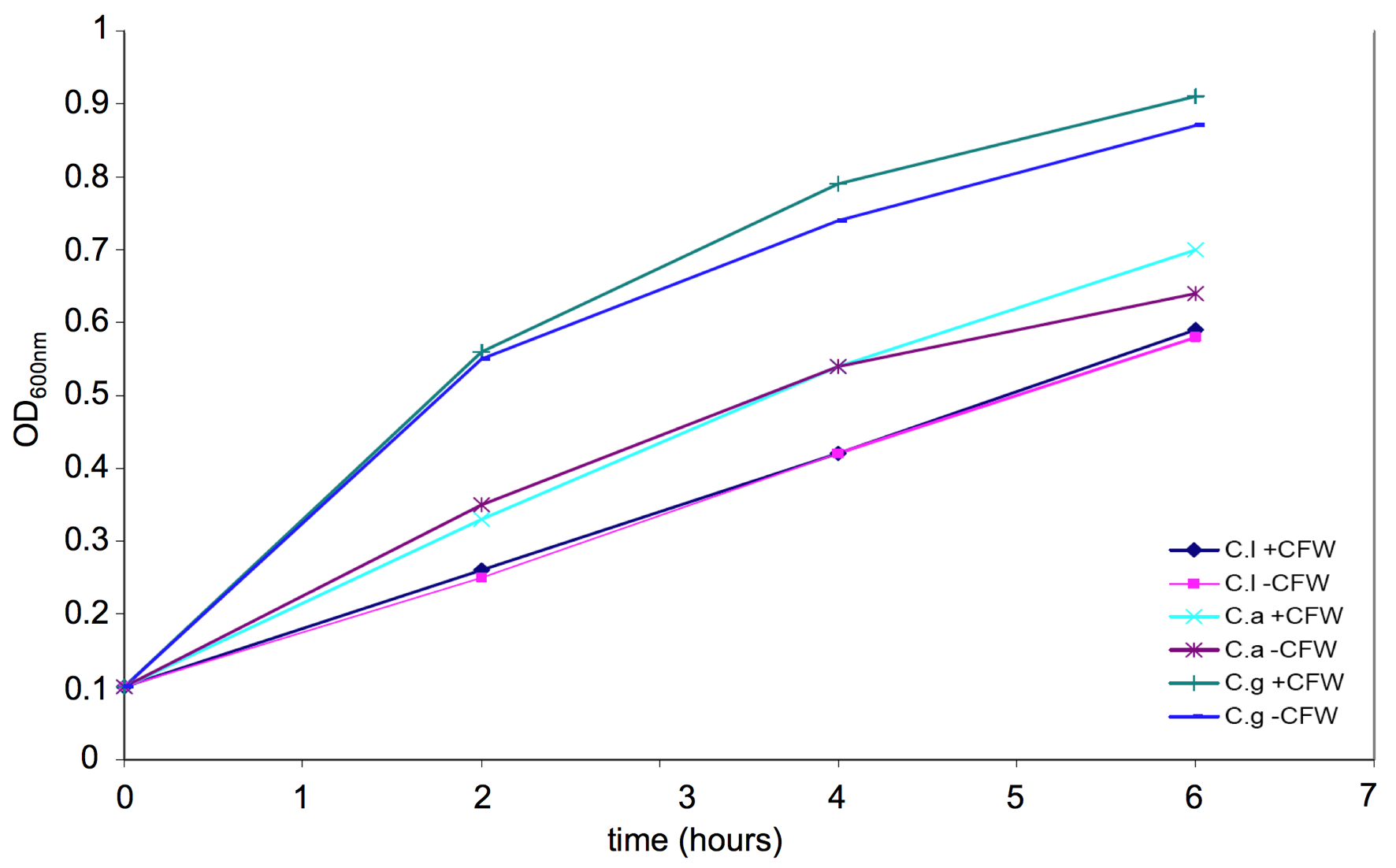

Supplement: Figure S1 — Yeast cells multiplication in cRPMI medium with and without CFW (5 µg/ml) by OD600nm measurements. C.a: C.albicans, C.g: C.glabrata, C.l: C.lusitaniae. (TIF) [file pone.0032621.s001.tif]

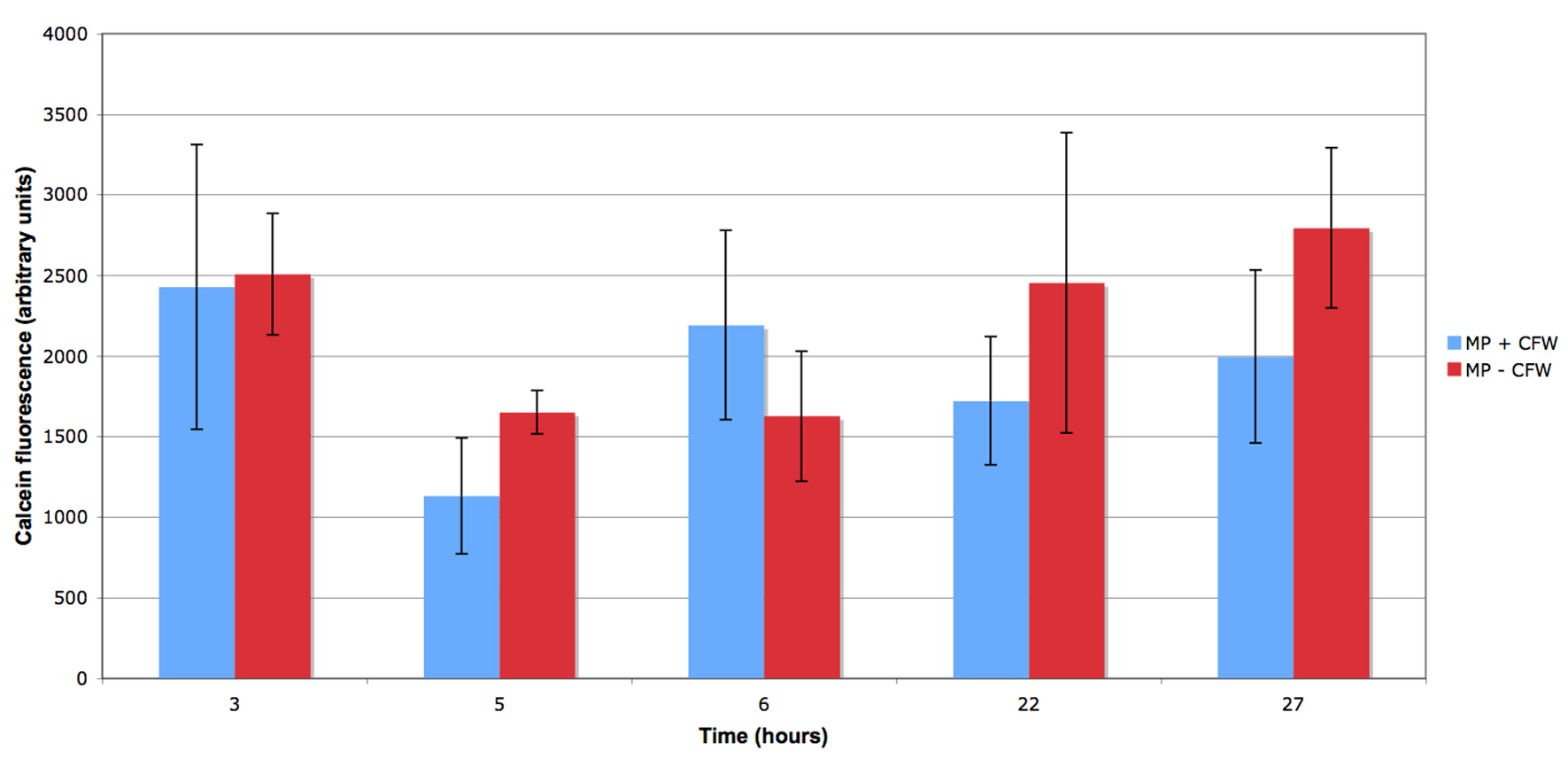

Supplement: Figure S2 — Macrophages viability in cRPMI medium with and without CFW (5 µg/ml) by calcein fluorescence measurements. Each bar is the average of two experiments ± standard error. (TIF) [file pone.0032621.s002.tif]

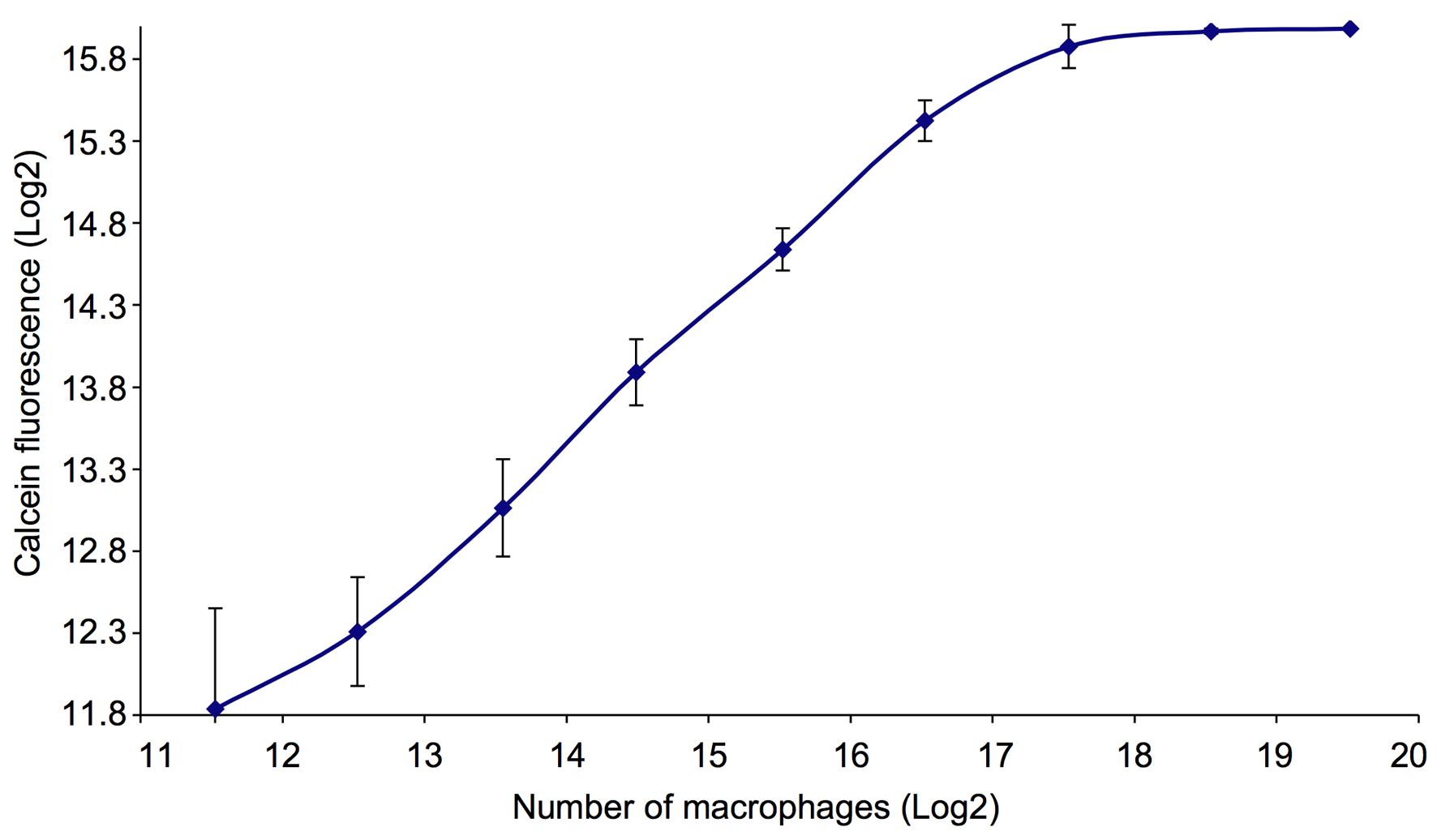

Supplement: Figure S3 — Calcein fluorescence varies proportionaly with the number of macrophages. Each bar is the average of three experiments ± standard error. (TIF) [file pone.0032621.s003.tif]

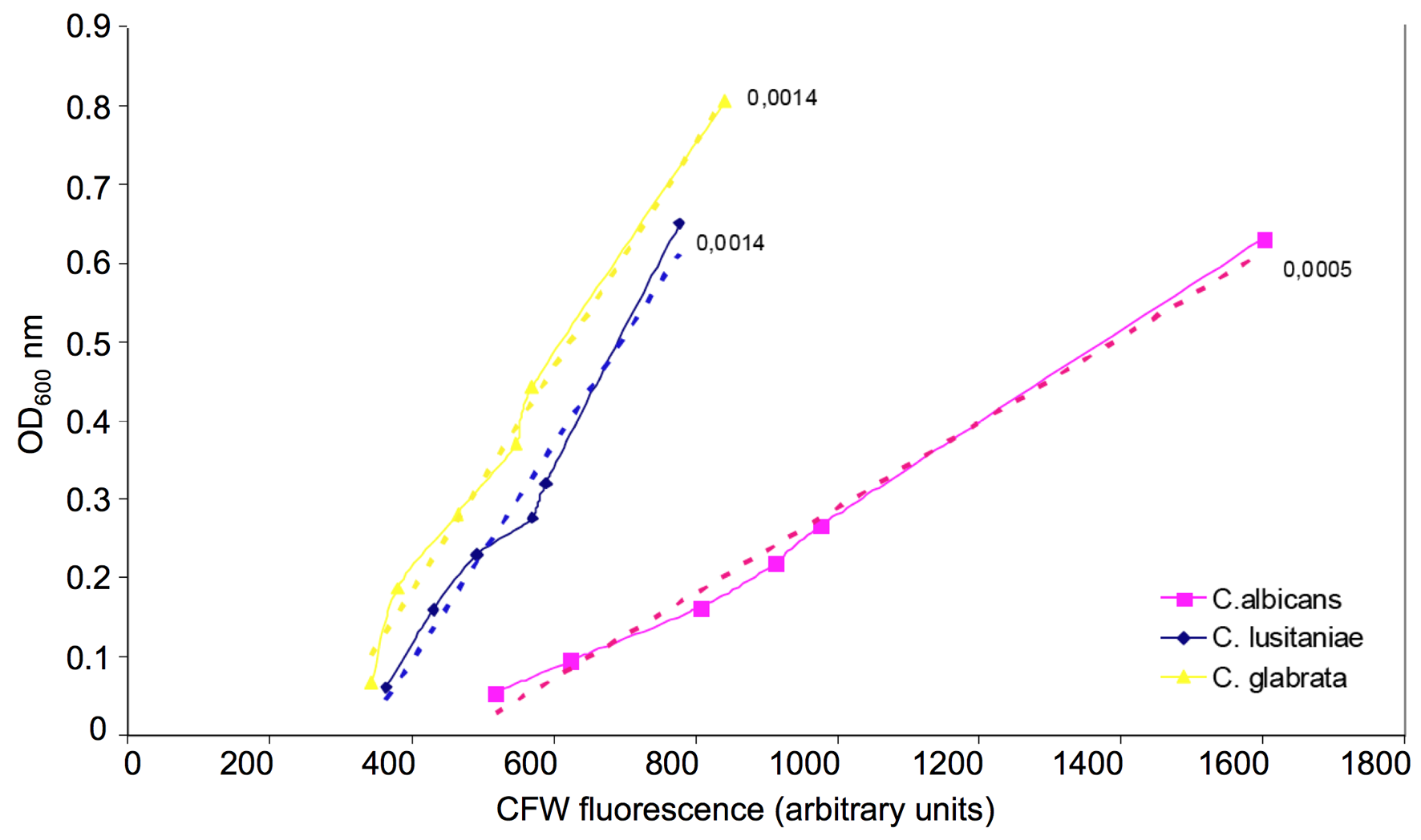

Supplement: Figure S4 — Yeast cells muliplication over time by OD600nm and CFW fluorescence measurements in cRPMI medium with 5 µg/ml of CFW. Dashed lines show linear regression lines, and their slopes are indicated. (TIF) [file pone.0032621.s004.tif]

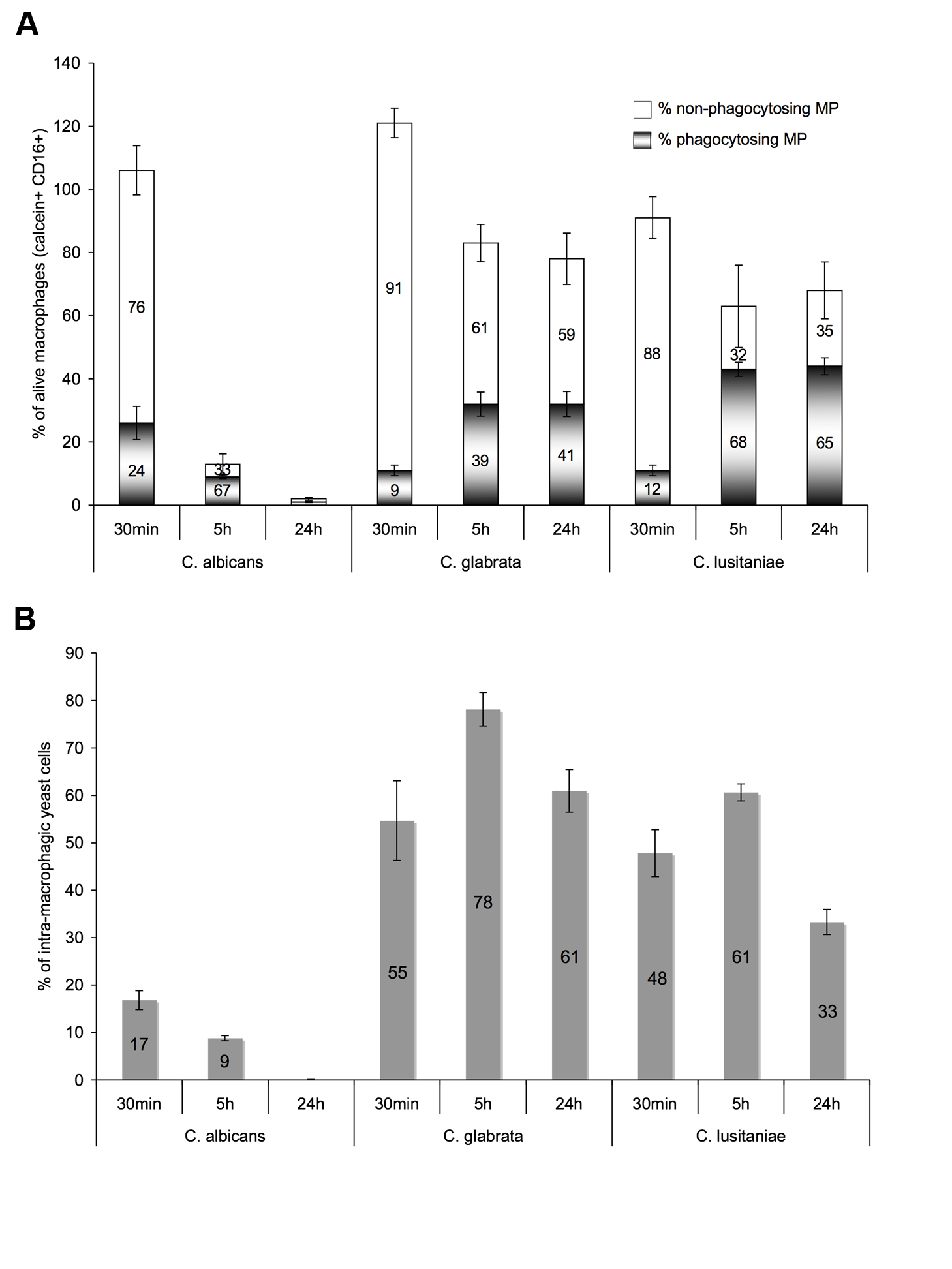

Supplement: Figure S5 — Analysis of the interactions involving the J774 macrophages and stationary-phase living yeast cells at 1M:1Y MOI over 24-hour time course experiments. Figure S5 details how the diagram of Figure 3A was built. Figure S5A shows the flow cytometry analysis of the macrophages and corresponds to the left part of the diagram of Figure 3A. Each bar represents the viability of infected macrophages compared to uninfected macrophages, and the numbers indicate the parts of phagocytosing (shaded tones) or non-phagocytosing (white tones) macrophages. Note that C. albicans engaged the higher part of macrophages in phagocytosis (T 30 min) and killed more macrophages (T 5 h and T 24 h) than C. glabrata and C. lusitaniae. Figure S5B shows the fluorometry analysis of the fungal cells and corresponds to the right part of the diagram of Figure 3A. Each bar represents the percentage of the total fungal biomass internalized in viable macrophages. Note the lower uptake of C. albicans cells. Each condition was performed in quintuplet (A) or in triplicate (B). Each bar is the average of three independent experiments ± standard error. (TIF) [file pone.0032621.s005.tif]

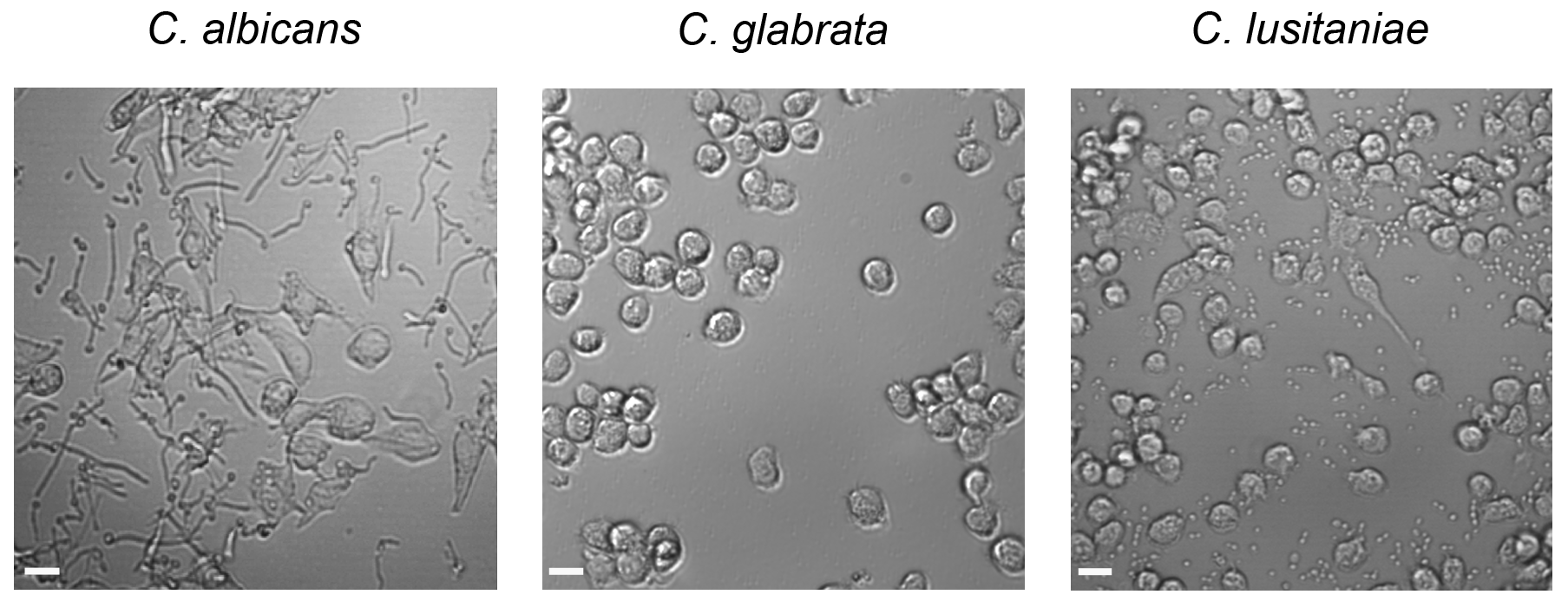

Supplement: Figure S6 — Representative pictures of the J774 macrophages after 5 hours of infection with the three Candida species in culture flasks at 1M:1Y MOI. Note that the totality of C. glabrata cells were engulfed, whereas C. albicans (mostly in filamentous form) and C. lusitaniae cells were still observed outside the macrophages. The scale bars represent 30 µm. See also Movie S1 showing the interaction of J774 macrophages with C. albicans at a MOI of 1M:1Y over a 5-hour incubation period. (TIF) [file pone.0032621.s006.tif]

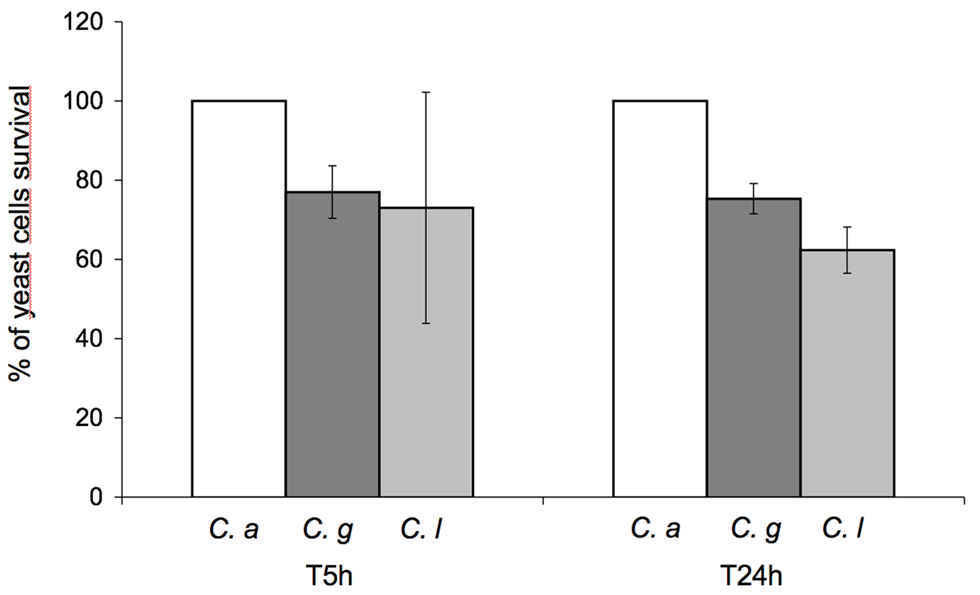

Supplement: Figure S7 — Macrophage fungicidal activity toward the different Candida species. To investigate the capacity of the macrophages to kill the different species of Candida, the survival of the yeast cells was determined following 5 and 24 hours of incubation with phagocytic cells at a 1M:1Y MOI (Method S2). 100% of the C. albicans cells survived, while 20% and 40% of the C. glabrata and C. lusitaniae cells were killed within 24 hours of interaction with the macrophages, respectively. C. a: C. albicans, C. g: C. glabrata, C. l: C. lusitaniae. (TIF) [file pone.0032621.s007.tif]
