# Supplementary material for: Development of an In Vitro Model for the Multi-Parametric Quantification of the Cellular Interactions between Candida Yeasts and Phagocytes
Source: PLoS One. 2012 Mar 30;7(3):e32621. doi: 10.1371/journal.pone.0032621 (PMC3316538; doi:10.1371/journal.pone.0032621)
Supplement: Method S1 — Method involved in glucose assay. See Table S1. (DOC) [file pone.0032621.s008.doc]

**Method S1.** **Glucose assay**. We used the Glucose (GO) Assay Kit (Sigma), according to the manufacturer’s instructions, to measure the availability of glucose during the infection experiments. Briefly, the intensity of the pink colored oxidized o-dianisidine proportional to the glucose concentration was measured at 540 nm. The J774 macrophages were infected as described previously by yeasts of the three *Candida* species at a MOI of 1M:1Y. The supernatants were collected after 5 and 24 hours of infection, centrifuged at 2000 x g for 5 min and filter-sterilized before the glucose assay was performed.
