# Supplementary material for: Development of an In Vitro Model for the Multi-Parametric Quantification of the Cellular Interactions between Candida Yeasts and Phagocytes
Source: PLoS One. 2012 Mar 30;7(3):e32621. doi: 10.1371/journal.pone.0032621 (PMC3316538; doi:10.1371/journal.pone.0032621)
Supplement: Method S2 — Method documenting the survival of ingested yeasts. See Figure S7. (DOC) [file pone.0032621.s009.doc]

**Method S2.** **Survival of ingested yeasts.** The infected macrophages were collected after trypsin treatment and centrifuged for 10 min at 10000 x g. Phagocytosed yeast cells were released by lysing the J774 macrophages in 1 ml of 0.1% ice-cold Triton X-100. As a control, yeast cells alone underwent the same treatment to verify that the Triton X-100 treatment did not alter yeast viability. The yeast cells were counted using a hemocytometer. To determine the survival of the yeast cells, 100 cells were plated on YPD plates in duplicate and incubated at 30°C for 24-48 h.
