# Supplementary material for: Development of an In Vitro Model for the Multi-Parametric Quantification of the Cellular Interactions between Candida Yeasts and Phagocytes
Source: PLoS One. 2012 Mar 30;7(3):e32621. doi: 10.1371/journal.pone.0032621 (PMC3316538; doi:10.1371/journal.pone.0032621)
Supplement: Table S1 — Macrophage cell death is not due to a nutritional depletion of the medium during infection. The quantity of glucose available was measured in the supernatant of the J774 macrophages that were infected with live or UV-killed yeasts of the three Candida species for 5 and 24 hours at a MOI of 1M:1Y (Method S1), and compared to uninfected macrophages, yeasts alone and fresh cRPMI alone containing 2 g/L glucose. The uninfected macrophages depleted approximately 40% of the glucose present in the media after 24 hours. When the macrophages were infected with the fungal cells, nearly 100% of the glucose was depleted after 24 hours, independently of the species used. After 5 hours, a higher glucose depletion was observed when the J774 cells were infected with C. albicans. Interestingly, C. albicans and C. glabrata alone used about 70–75% of the glucose at T 5 h, whereas C. lusitaniae used only 40%. Next, we checked if this glucose depletion could be responsible for macrophage mortality during infection (data not shown). The macrophages were incubated for five hours with the different supernatants collected from infection experiments and filter-sterilized, before assessing their survival by flow cytometry. Despite the total depletion of glucose in the media after 24 hours of infection, none of the supernatants tested triggered macrophage cell death. These results indicated that J774 macrophage mortality was directly related to the phagocytosis of the Candida cells. (DOC) [file pone.0032621.s013.doc]

**Table S1.** Relative concentration of glucose remaining in the supernatant of cultures of different cell types

| Supernatants of | 5h^a^ | 24h^a^ |
| --- | --- | --- |
| cRPMI alone | 100 | 100 |
| J774 alone | 80 | 64 |
| *C. albicans* alone | 24 | 0 |
| *C. glabrata*  alone | 31 | 0 |
| *C. lusitaniae* alone | 56 | 0 |
| J774 + live *C. albicans* | 17 | 0 |
| J774 + live *C. glabrata* | 78 | 6 |
| J774 + live *C. lusitaniae* | 72 | 0 |
| J774 + UV-killed *C. albicans* | 88 | 16 |
| J774 + UV-killed *C. glabrata* | 72 | 13 |
| J774 + UV-killed *C. lusitaniae* | 74 | 19 |

^a^ Relative % of glucose compared to cRPMI alone containing 2 g/L glucose.
